# S209. PATIENT EXPERIENCES OF THE EARLY SIGNS ACTION PLAN

**DOI:** 10.1093/schbul/sby018.996

**Published:** 2018-04-01

**Authors:** Nils Sjöström, Maria Genberg, Alexander Marteleur, Eva Andreasson, Margda Waern

**Affiliations:** 1 Sahlgrenska University Hospital; 2 Region Västra Götaland; 3 Sahlgrenska University Hospital, University of Gothenburg

## Abstract

**Background:**

Coercive psychiatric care in Sweden has been criticized by the UN and alternative therapies are called for upon from Swedish politicians. The Early Signs Action Plan was developed to reduce force and promote cooperation between patients and their healthcare providers.

**Aim:**

Describe the patients’ experience of care when the Early Signs Action Plan is activated in connection with an exacerbation of psychotic illness.

**Methods:**

Qualitative research study. Semi-structured interviews (anticipated N=10) will be conducted with patients for whom Early Signs Action Plans were activated. Interviews are recorded and transcribed verbatim. Content analysis is used to analyze the data.

**Results:**

Preliminary results from the first five interviews suggest that the action plan facilitates shared decision making and encourages safety measures, and compulsory inpatient care can thus be avoided. The results from the entire study will be presented at the Conference.

**Discussion:**

Preliminary findings suggest that the Early Signs Action Plan seemed to be a useful tool to im-prove patient participation and reduce the need for compulsory inpatient care when exacerbations occur.

